# Workforce Trends Among Canadian Medical Oncologists and Medical Oncology Trainees over Two Decades

**DOI:** 10.3390/curroncol32020070

**Published:** 2025-01-28

**Authors:** Adam Fundytus, Sarah Cook, Steven M. Yip, Shaun K. Loewen, Desiree Hao

**Affiliations:** 1 Department of Oncology, British Columbia Cancer Agency, Victoria, BC V8R 6V5, Canada; 2 Division of Medical Oncology, Arthur JE Child Comprehensive Cancer Centre, Calgary, AB T2N 4N1, Canada; 3 Cumming School of Medicine, University of Calgary, Calgary, AB T2N 2T8, Canada; 4 Division of Radiation Oncology, Arthur JE Child Comprehensive Cancer Centre, Calgary, AB T2N 4N1, Canada

**Keywords:** medical oncology, health human resources, workforce

## Abstract

**Background:** Understanding oncology health human resources across Canada is critical to the delivery of quality cancer care. Little has been published about the medical oncology (MO) workforce and trainees; this study sought to characterize trends in the MO workforce and explore the relationship between medical oncologists and cancer incidence as a surrogate demand marker. **Materials and Methods**: Publicly available databases from the Canadian Medical Association, the Canadian Institute of Health Information, and the Canadian Post MD Education Registry were utilized to estimate the number, demographics, and regional distribution of practicing MOs and MO trainees between 1994 and 2020. Cancer incidence by province was obtained from Statistics Canada. To estimate changes in demand for, and supply of, medical oncology services over time, annual cancer incidence to MO provider ratios were calculated. **Results:** Between 1994 and 2020, annual cancer incidence nationally rose from 120,255 to 225,800 cases, while the number of MOs increased by 298%. Incident cancer case to medical oncologist (MO) ratio dropped from 749:1 to 352:1 in the same time. However, the MO workforce is aging; in 2020, 40% of providers were ≥50 years old versus 24% in 1994. Trends in Canadian MO trainees mirror MO trends. Ontario has the largest proportion of the country’s MOs (34% in 2020) and MO trainees (49%). **Conclusions:** Although the Canadian MO workforce has grown, more MO providers are nearing retirement age, which may influence future workforce trends. Ongoing monitoring of human resources in oncology is essential to ensure future demands for services are met.

## 1. Introduction

Cancer is the leading cause of death in Canada, accounting for 27% of all national deaths at the time of the last national census in 2023 [1]. Additionally, cancer rates have risen annually in Canada, with 239,100 cases projected for the year 2023 [2]. The rising burden of cancer within Canada is of interest to health system decision makers. Global projections predict a rise in both the incidence of cancer as well as the demand for systemic therapy in upcoming years [3,4]. Medical oncologists are physicians who manage the care of cancer patients and oversee the delivery of systemic therapy and, as a whole, they can be described as the MO workforce. Supply and demand projections from America, Europe, Australia, and Spain suggest that the demand for oncologic services may outstrip the supply if adequate workforce growth is not maintained [4,5,6,7]. Thus, from a policy perspective, it is vital that trends in the MO workforce within Canada are summarized such that prior policy can be evaluated and future policy directed in order to ensure an adequate supply of MOs to service the country’s cancer system.

Although workforce trends for radiation oncologists in Canada have been published [8], no readily available summary of the Canadian MO workforce has been performed. An annual headcount from the Canadian Medical Association (CMA) [9] provides an overview of the MO workforce nationally in a given year but with limited examination of longitudinal trends and regional data [10] and, additionally, there is no information about demographics trends of MO trainees (residents and fellows) over time, which is key to informing future modeling of workforce supply. Recent work predicting future MO workforce requirements within Canada focused on the number of MOs in the country as a whole without acknowledging that there are likely regional differences in MO supply and, thus, possible regional shortfalls, which would need to be addressed for optimal cancer care [10]. Importantly, this work did not include any analysis on the supply of trainees into the MO system, which directly affects the future workforce. Therefore, we aim to collate available data on the MO workforce in Canada and to perform a descriptive analysis of this workforce over a 25-year span. Here, we describe the changes in the composition of the existing Canadian MO workforce and the supply of future MOs (trainees) nationally and by region between 1994 and 2020. A secondary objective was to characterize MO workforce metrics relative to annual cancer diagnoses over time as a surrogate of cancer burden relative to provider coverage.

## 2. Materials and Methods

### 2.1. Study Design

This study is a descriptive analysis of pre-existing databases. Because no hypothesis testing was conducted and the purpose of this analysis was to serve as a summary of the available data, no statistical analysis was performed.

### 2.2. Databases for MO Workforce

Information on practicing MO was obtained from both the Canadian Medical Association—Physician Data Centre (CMA-PDC; 1994–2019) [9] and the Canadian Institute for Health information—Scott’s Medical Database (CIHI-SMDB; 1994–2020) [11]. Details of each database used are shown in Table 1. Data from each database were manually extracted by one reviewer (AF). MO workforce data were extracted for the years 1994–2020 because these years included the greatest overlap between the CIHI-SMDB database (1968–2020) and the CMA-PDC database (1994–2019). Each database reports similar demographic data including the number of MO practicing in each province, as well as their gender and age. For categories where both databases contained identical information such as the number of MO, the data were averaged. The age distribution of MO was categorized using different cutoffs in the CIHI-SMDB versus CMA-PDC databases; thus, data could not be aggregated and were instead reported separately for each database.

### 2.3. Database for MO Trainees

Information on MO trainees (1994–2020) was derived from the Canadian Post M.D Education Registry (CAPER) database [12]. The CAPER database collects data submitted by the post-graduate programs of all 17 Canadian medical schools and includes two-year residents as well as fellows undergoing additional training after graduation from medical oncology residency programs. The 1994–1999 reports were obtained from CAPER and added to the CAPER subspecialty reports for 2000–2019, which are available online. Our analysis excludes visa-sponsored trainees who are required to return to their home country after completion of training. The data are tabulated per academic year (July–June of the following year).

### 2.4. Regional Analyses

Regional analyses were performed according to the official regions of Canada [14]. Canada is divided into the following regions: (1) West Coast (British Columbia); (2) Prairie Provinces (Alberta, Saskatchewan, and Manitoba); (3) Central Canada (Ontario and Quebec); and (4) Atlantic Canada (Newfoundland, Nova Scotia, New Brunswick, and Prince Edward Island). As Central Canada contains the two most populous Canadian provinces (Quebec, and Ontario), separate analysis were performed for each province. The Northern Territories were not included in the analysis since there are no MO or training programs in these regions.

### 2.5. Cancer Incidence

National and regional cancer incidence data for 1994 to 2020 were obtained from the Canadian Cancer Registry database with Statistics Canada. Projected cancer incidence data were derived using CANPROJ projection software [15]. CANPROJ is a validated projection modeling package in R software (version 4.1.0, R Foundation for Statistical Computing, Vienna, Austria and RStudio version 1.4.1717 RStudio Inc., Boston, MA, USA) that uses trends in historical data to select the best-fit model for projected years based on a decision algorithm comprised of six age–period–cohort models [15]. Since cancer incidence data for the province of Quebec were not available from 2011 onwards, Quebec-specific incidence counts were projected from 2011 to 2020 versus 2020 only for all other regions [16]. Annual ratios of incident cancer cases per medical oncologist were calculated using the number of actual or projected incident cancer cases in Canadians 20–90 years old divided by the number of practicing medical oncologists from 1994 to 2020. Cancer incidence in Canadians 0–19 years old was excluded because the care of cancer patients who are under 18 years old are managed by pediatric oncologists. Both national and regional incident cancer cases per medical oncologist ratios were generated.

## 3. Results

### 3.1. National Demographic Trends for MO

**Figure 1:**
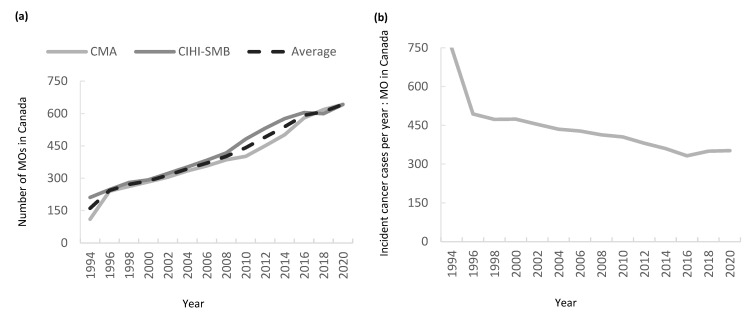

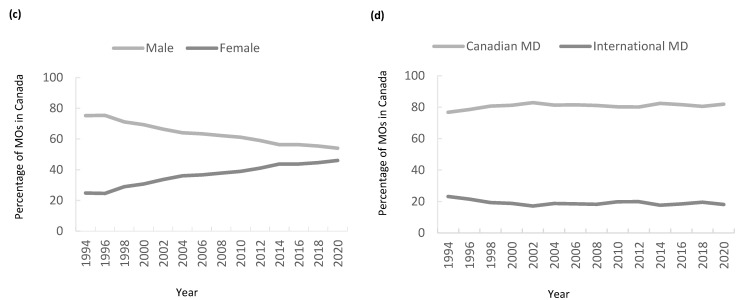
(**a**) Absolute number of medical oncologists (MO) in Canada between 1994 and 2020; (**b**) incident cancer cases per MO in Canada 1994–2020; (**c**) percentage of MO in Canada by gender; (**d**) percentage of MO in Canada by medical school training location.

Between 1994 and 2020, the number of medical oncologists in Canada rose from 161 in 1994 to 642 in 2020, an absolute increase of 481 MOs (+299%) (Figure 1a). The average annual growth rate of the MO workforce over the period was 5.7% per year, with a maximum increase of 39.9% between 1994 and 1995 (Figure 1a). The minimum rate of growth of 1% occurred between 2017 and 2018. Over the study period, the number of incident cancer cases in Canada rose by 88% from 120,255 in 1994 to 225,800 in 2020. The incident cancer cases per medical oncologist ratio nationally fell from 749:1 to 352:1 (Figure 1b). The proportion of female oncologists in Canada rose from 24.9% in 1994 to 46.0% in 2020 (Figure 1c). The proportion of Canadian MO attaining their medical degree in Canada was stable over time, with 77% and 82% of MO attaining their MD in Canada in 1994 and 2020, respectively (Figure 1d).

**Figure 2:**
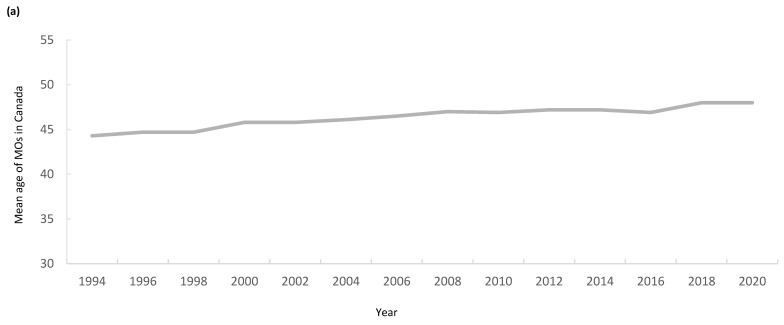

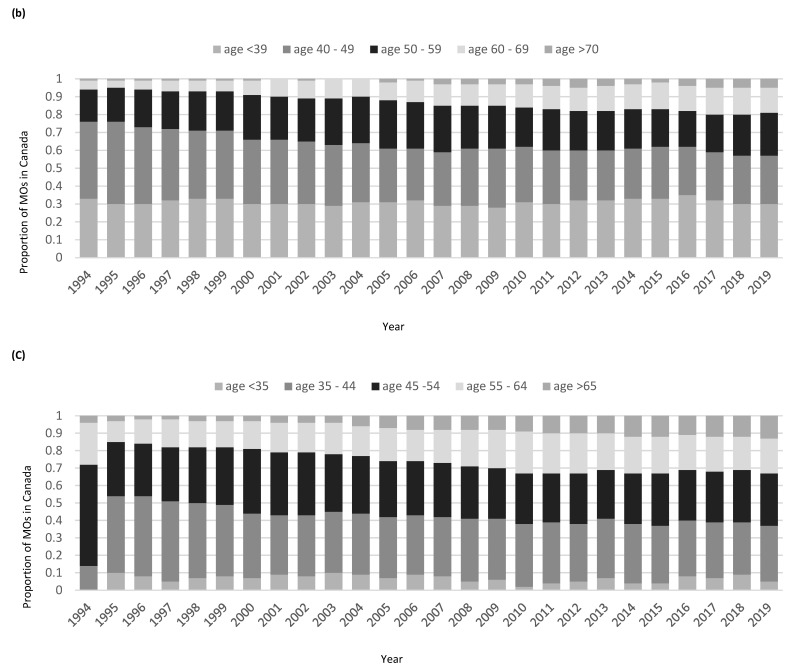
(**a**) Average age of Canadian MO workforce by year; (**b**) proportion of MO in each age category over time (CIHI); (**c**) proportion of MO in each age category over time (CMA).

The average age of the MO workforce in Canada rose from 44 years old in 1994 to 48 in 2020. (Figure 2a). The proportion of oncologists in each age category over time as estimated by each database are depicted in Figure 2b (CIHI-SMDB) and Figure 2c (CMA-PDC). In 1994 and 2020, the CIHI proportion of oncologists <39 in age was similar at 33% and 32%, respectively; however, the proportion between age 40 and 49 fell from 44% to 28%, while the proportion between 50 and 64 rose from 22% to 29%. Only 2% of oncologists were over 65 in 1994 and, by 2020, this number had risen to 11%. The CMA-PDC age data show a similar trend, with the proportion of oncologists aged 55 years old or greater rising from 15% in 1994 to 31% in 2019 (Figure 2c).

### 3.2. Regional Demographic Trends for MO

**Figure 3:**
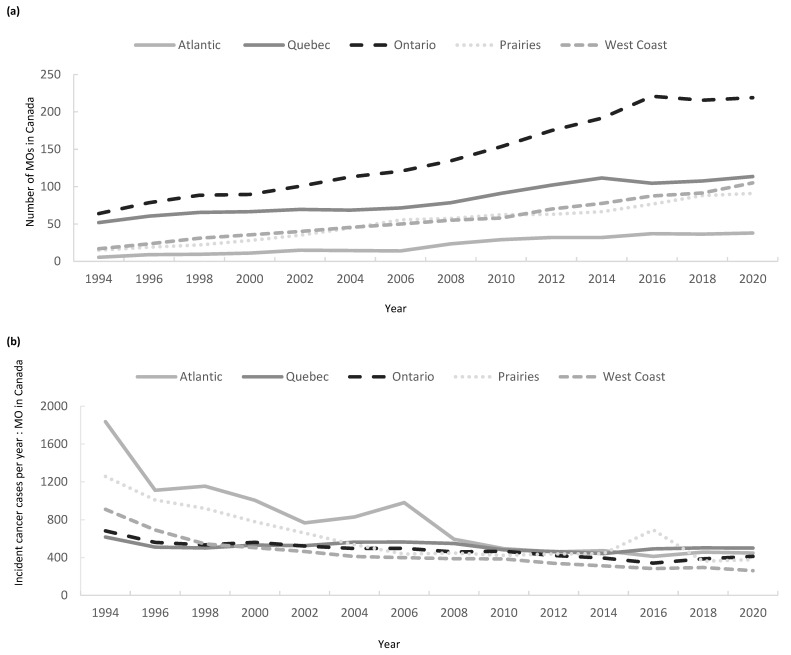

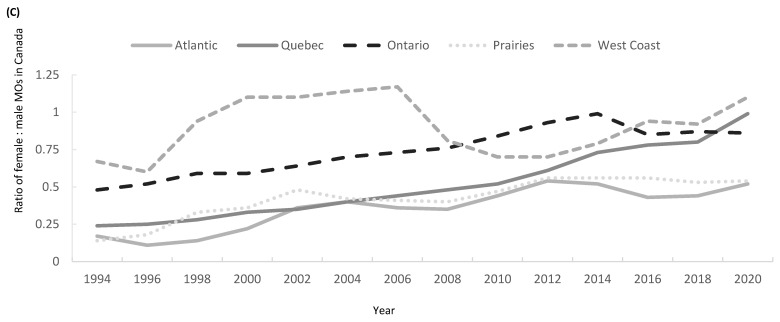
(**a**) Number of medical oncologists (MO) across Canada between 1994 and 2020 by region; (**b**) ratio of incident cancer cases per MO provider; (**c**) ratio of female/male MO across Canada, 1994–2020, by region.

The number of MO increased across all regions of Canada over the study period (Figure 3a). The largest proportional increase was seen in the Atlantic provinces, with 6 MO in 1994 compared to 38 in 2020 (+533%), followed by the West Coast (17 to 105:+518%), Prairie Provinces (15 to 91:+507%), Ontario (64 to 219:+242%), and Quebec (59 to 189:+220%).

The increase in the number of MOs in all regions was higher over the first half of the study interval (1994–2007) versus the latter half (2008–2019) (Figure 3a).

Between 1994 and 2020, the ratio of incident cancer cases per MO provider fell across all regions (Figure 3b). In 1994, the Atlantic and Prairie Provinces had higher incident cases per MO ratios than the other regions, with ratios of 1836:1 and 1257:1, respectively, compared to 909:1 on the West Coast, 682:1 in Ontario, and 543:1 in Quebec. In 2020, the highest ratio was in Atlantic Canada (447:1), followed by Ontario (411:1), the Prairie Provinces (377:1), Quebec (300:1), and the West Coast (261:1). Atlantic Canada is the only region that experienced repeated peaks in this ratio, with specific maximums seen in 1998 (1154:1) and 2006 (980:1) after sustained periods where the ratio fell consistently (Figure 3b).

According to CIHI-SMDB for the year 2020, Atlantic Canada has the lowest proportion of female MO (34%), followed by the Prairie Provinces (35%), Ontario (46%), Quebec (50%), and the West Coast (52%) (Figure 3c). The proportion of female MO providers increased in all regions between 1994 and 2020.

**Figure 4:**
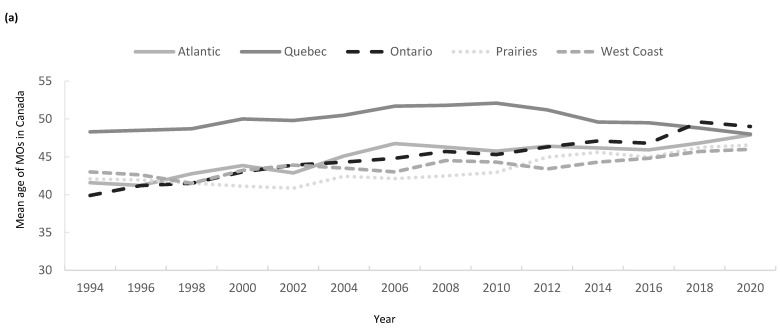

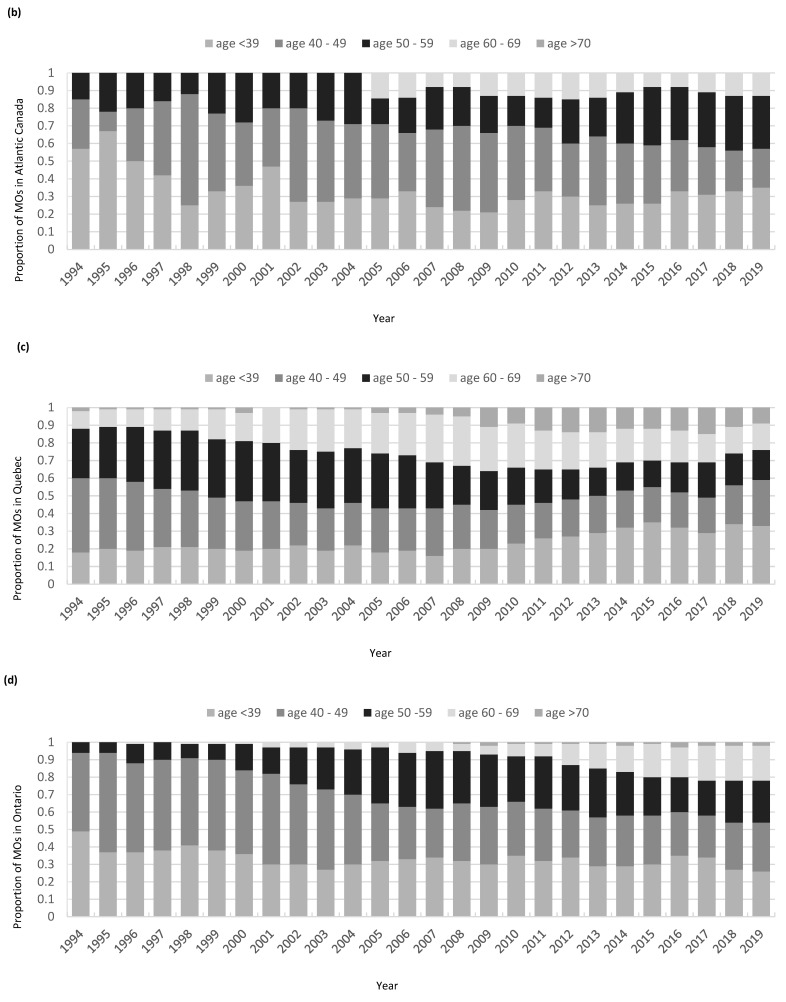

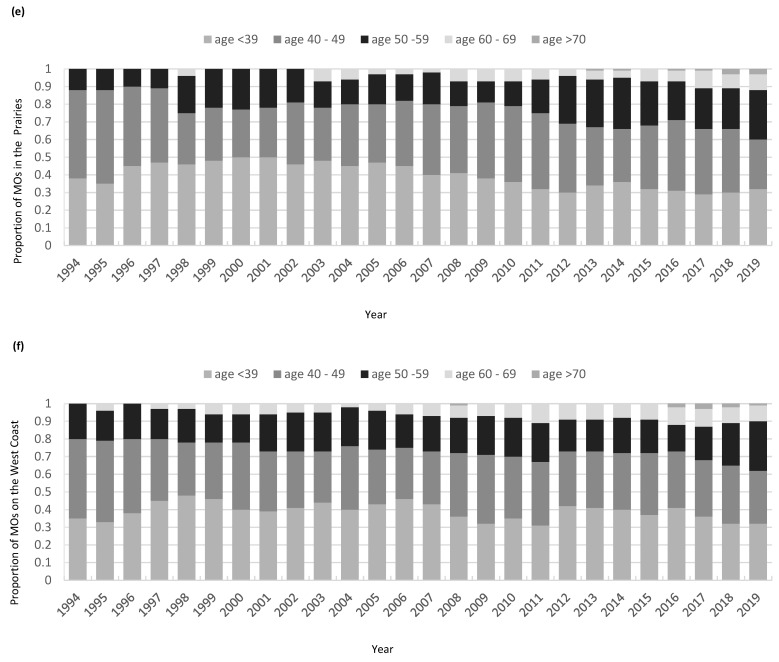
(**a**) Mean age of MO across Canada by region; proportion of MO by age category in (**b**) Atlantic Canada, (**c**) Quebec, (**d**) Ontario, (**e**) the Prairies, and (**f**) the West Coast.

Regional variation in the age composition of oncologists according to CIHI-SMB is shown in Figure 4 for all regions. In 1994, the average age of an MO was highest in Quebec (48 years) compared with the West Coast (43), Atlantic Canada (42), the Prairie Provinces (42), and Ontario (40), which had the lowest average age. In contrast, in 2020, the average age of MO was the highest in Ontario (49 years), followed by Atlantic Canada (49 years), Quebec (48 years), the Prairie Provinces (47 years), and the West Coast (46 years).

### 3.3. Medical Oncologist Trainees

**Figure 5:**
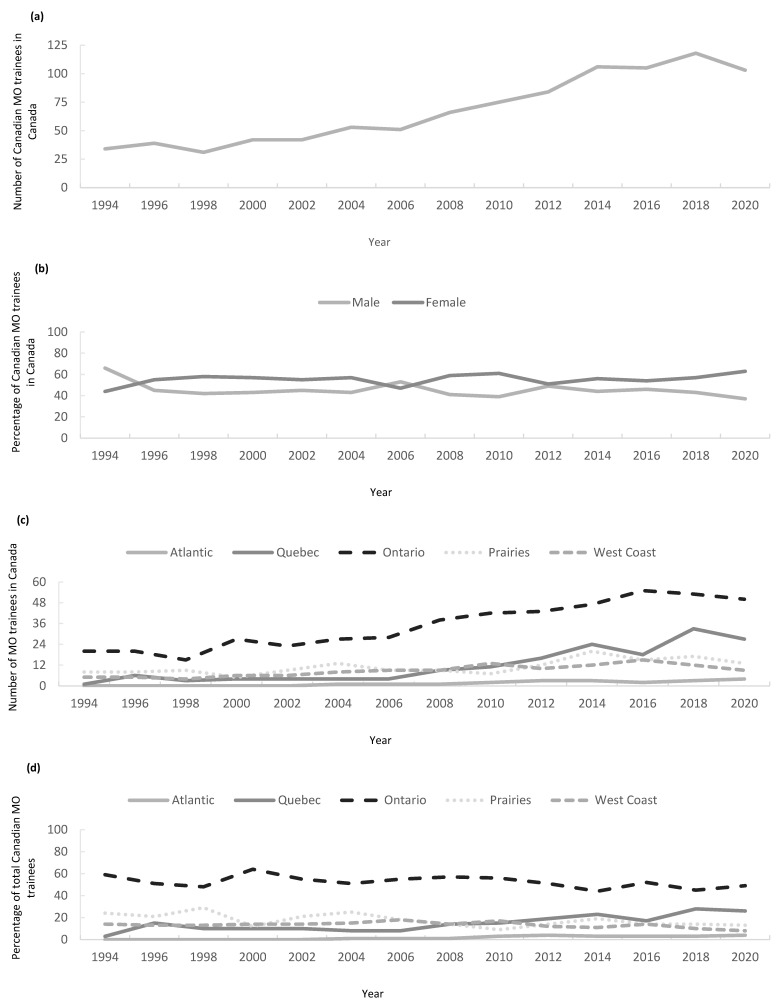
Canadian medical oncology trainee trends between 1994 and 2019. (**a**) Number of Canadian citizen/permanent MO trainees; (**b**) gender proportion of medical oncology trainees; (**c**) number of medical oncology trainees by region; (**d**) proportion of total Canadian medical oncology trainees trained in each province per year.

The number of MO trainees in Canada rose from 34 in the 1994–1995 academic year to 99 in the 2019–2020 academic year, representing a 191% increase (Figure 5a). These trainees were trained at nine universities in four provinces (British Columbia, Alberta, Ontario, and Quebec). With time, MO training programs emerged at the University of Manitoba (Manitoba) in 1993/94, University of Laval (Quebec) in 1995/96, Dalhousie University (Nova Scotia) in 2003/04, Queens University (Ontario) in 2006/07, University of Sherbrooke (Quebec) in 2010/11, and Memorial University (Newfoundland and Labrador) in 2015/16, increasing training capacity to 15 residency programs in seven provinces. No MO training programs exist in Saskatchewan, New Brunswick, Prince Edward Island, and the Canadian Territories.

The proportion of MO trainees by gender is shown in Figure 5b. Female trainees outnumbered male trainees for all years except for 1994–1995 and 2006–2008 (Figure 5b). In 1994, 66% of trainees were male compared to 42% in 2019, corresponding to a shift towards consistently more female MO trainees throughout the study period.

The regional supply of trainees through time is shown in Figure 5c. The raw number of trainees has risen across all regions, but the proportional increase varies by region. The proportion of total Canadian MO trainees supplied by each province is shown in Figure 5d. Only 1 trainee per year came from Quebec in 1994 (3% of total trainees) but the number increased to 18 (18% of total trainees) in 2019, a rise of 1700%. Atlantic Canada trained four MO trainees (4% of total) in 2019 compared with none in 1994. While the proportion of total MO trainees trained in Ontario has fallen between 1994 (56%; 20 trainees) and 2019 (49%; 49 trainees) the absolute number has risen. Similarly, the proportion of MO trainees trained on the West Coast has also fallen between 1994 (15%; 5 trainees) and 2019 (12%; 12 trainees). The Prairie Provinces have maintained the same portion of national trainees between 1994 (15%; 5 trainees) and 2019 (16%; 16 trainees) while increasing the absolute number of trainees.

## 4. Discussion

This is the first report describing temporal trends in the Canadian MO workforce, including the MO trainee cohort, by age, gender, and region over a 25-year period. Several important findings have emerged. Between 1994 and 2019, the Canadian MO workforce has increased by 298%, a pace exceeding the rise in cancer incidence. Regional differences in the supply of medical oncologists relative to regional cancer incidence were evident, with Atlantic Canada persistently having the lowest MO supply relative to its annual cancer incidence. Over time, the average age of Canadian MO has increased and, in Ontario, a larger proportion of MO are nearing retirement age than other provinces.

The rise in the number of incident cancer cases relative to MO providers has resulted in a lower ratio of annual incident cancers per MO provider over time, from 749:1 in 1994 to 352:1 in 2020. These data could be explained by either the appropriate expansion of the MO workforce to accommodate increased demand or initial undersupply of MO relative to demand, followed by a supply correction. To determine whether Canada at present has an undersupply of oncologists relative to its cancer incidence, we compared Canada’s incident cancer/MO ratio to that of other health systems across similar developed countries (Table 2) [4,17]. Canada’s ratio of incident cancer per MO provider remains higher than the United States, Australia, and most European comparators [4,17]. Although the number of MO in Canada has increased with time, comparative data suggest a relative undersupply persists. What is perhaps most concerning is that, although Australia has a higher supply of MO compared with our estimates, a recent study from Australia suggests that even their workforce is inadequate to meet current oncologic demand based on Australian providers seeing higher than expected new consult numbers per year [18]. Empiric evidence of Canadian MO undersupply is shown by recent reporting, suggesting significant delays in receiving chemotherapy in both the West Coast and Prairie regions of Canada [19,20].

Characterizing growth in the MO workforce may not accurately quantify workload per MO. Estimates suggest that 57% of all incident cancer will require systemic therapy; however, this remains the most used workforce metric across the literature; thus, this is the metric we examined [3]. MO workload is influenced by complex factors, including referral rates, cancer incidence rates in each jurisdiction, treatment complexity, potential inpatient care for treatment toxicity or complications, and participation in clinical trial or medical education activities [5,6,21]. A recent Canadian projection model has suggested that, even if the number of Canadian MO increases with time relative to cancer incidence, the number of systemic therapy starts per oncologist is still projected to rise and patients are receiving more lines of treatment than in the past [10]. Therefore, cancer incidence and consultation workload are imperfect surrogates for provider workload and more robust metrics are needed. This is further supported by the fact that waitlists on the west coast are currently far in excess of acceptable standards for Canada; yet, according to our study, they have the most favorable incidence to MO ratio and, thus, we would expect to see the lowest wait time in this region [20]. Therefore, updating the Canadian Oncology Utilization study, completed in 2002, would be of value, as many of the assumptions from this study are unlikely to retain validity 20 years later [22].

Comparing our findings with Canadian Radiation Oncology (RO) workforce data [8], there were no notable peaks in the ratio of cancer incidence per provider among MO as there were in RO, which rose to a peak ratio of 1196:1 in 2003/2006. Drivers of this peak were multifactorial but may have included large swings in the number of Canadian RO trainees coinciding with a large number of retirements in Quebec and an exodus of trainees due to poor job market conditions between 1996 and 2001 [8]. This underscores the importance of a stable trainee supply if we are to maintain a stable oncology workforce and avoid the undersupply issues seen in radiation oncology.

Regionally, Atlantic Canada appears to have a relative undersupply of oncologists compared with the rest of the country. In 2020, Atlantic Canada had the highest ratio of annual incident cancer cases per MO provider at 447:1 and the lowest ratio with 261:1 in BC. This trend parallels the RO literature, with Atlantic Canada having a higher proportion of the nation’s cancer cases than they have of the countries ROs [8]. The reasons for this are not entirely clear but warrant further exploration.

Aside from Quebec, there is a trend towards an older demographic, with a larger proportion of MO now in the age bracket >50 years of age across Canada. For example, in Ontario, 44% of MO are now >50 years old, with 14% now over the age of 65 years compared with 6% and none in 1994, respectively. As more MOs reach the typical retirement age of 65 [23], increased turnover and replacements will be required to maintain stability in the workforce. In Quebec, the average age of MO rose rapidly in 1994 when many practicing hematologists recertified as medical oncologists, which, together with low trainee numbers, led to the Quebec MO population being older than the rest of Canada. More recently, the proportion of Quebec MOs < 50 years of age has started to rise (Figure 2), as the proportion of Canadian MO trainees from Quebec has risen from 2/26 (8%) in 1994 to 18/99 (18%) in 2019. These data support the assertion that workforce numbers can potentially be modulated through increases in the number of trainees in each region.

We observed a trend towards gender parity over time in the MO population. As of 2020, 46% of medical oncologists were female compared with only 25% in 1994. However, this proportion varied by region, with only 34% and 35% of MO in Atlantic Canada and the Prairies, respectively, being female in 2020. Our findings mirror a sample of Australian MO from 2018, which showed that 49% of survey respondents were female [24]. Prior qualitative evidence suggests the emergence of a perception that female MO are becoming more common and that the specialty itself is changing to accommodate the needs of gender balance [25]. Specifics as to what this accommodation means are not given in this paper.

Between 1997 and 2001, there was an apparent contracture in MO residents, with only 39, 37, 31, 37, and 42 residents in each year, respectively. Ontario alone had 17 MO residents in 1996, but this fell to 13, 10, 17, 14, and 16 for the ensuing five years. Although the number of Canadian MOs expanded during this time, the population of MOs in Ontario declined slightly, hitting a trough in the year 2000. A similar trend is observed in Quebec between 1994 and 2008, where trainee numbers are proportionately lower compared with the rest of Canada. The stagnant MO trainee numbers may reflect healthcare cuts in the mid-1990s across multiple jurisdictions, including Quebec [26]. Anecdotally, our colleagues in Quebec have endorsed a period ~1998–2008 where medical school enrollment was actively cut, leading to a subsequent decline in subspecialty training. Following reversal of these spending cuts, there was both a rapid increase in the proportion of trainees from Quebec after 2012 and the slope of the MO supply curve thereafter (Figure 2 and Figure 5).

Several policy implications arise from our study. First, Canada appears to have a relative undersupply of medical oncologists relative to many other developed health systems. Thus, supply side measures to increase the number of MO would be beneficial. Theoretically increasing the number of oncology training slots should help to increase the net supply of oncologists in Canada. However, a look at the 2025 Canadian Residents Matching Service (CaRMs) subspecialty match report suggests that there were unfilled MO residency slots in all of our described regions [27]. Addressing this supply side shortfall will require a better understanding of the underlying causes for the shortfall and strategies targeting the specific issues identified. For instance, the number of domestic trainees remains low, policies to facilitate recruitment of more foreign trained MOs may help address wait times.

Our study has several limitations. Most importantly, the numbers reported by the two major databases used are not entirely concordant from year to year. Thus, the exact number of active MO in the country is not precisely known, which means these data should not be used for granular policy decisions such as local hiring of MO. Additionally, both databases may overestimate the number of practicing MO in a region. For example, the number of departures from the MO workforce are not reliably captured by either database; counting oncologists who have retired or left the country in our sample could over-estimate the supply of MO in Canada for a given year. Additionally, neither database captures the full-time equivalent (FTE) an individual MO works nor part-time employment, making it challenging to ascertain the full capacity of the workforce. Furthermore, the main metric of annual incident cancer per MO provider is only a crude estimate of the relationship between supply and demand. This metric only takes into account new cancer cases but does not account for cancer prevalence, referral rates, surveillance for cancer recurrence or progression, treatment complexity, and risk of complications, all of which contribute to the clinical workload in practice. Finally, we did not report the number of dedicated fellowship positions per region. Theoretically, provinces with more fellowship slots may ultimately retain a higher proportion of trainees than provinces whose residents transition to fellowship in other provinces, further exacerbating regional differences in MO supply. To improve workforce modelling accuracy in the future, an integrated database which includes both individual oncologist workload metrics (annual consults, follow-up assessments and treated patients) as well as region-specific cancer incidence alongside the number of oncologists in each region would be extremely useful. Additionally, continuing this work in 5-year windowing would allow for ongoing monitoring of oncology workforce capacity and inform policy pertaining to health human resource recruitment and funding.

## 5. Conclusions

Our study shows that the number of medical oncologists in Canada has increased over a 25-year period, despite which the ratio of incident cancer/MO is still higher in Canada compared with other developed countries. Furthermore, our MO population is aging, which may have significant implications for the MO supply in the future. Since the number of trainees produced by a region directly influences the number of practicing MO, more MO trainees in underserved regions such as Atlantic Canada may help address undersupply in those regions of Canada. Future studies should also consider examining alternate models of care, such as general practitioners in oncology (GPOs), nurse practitioners (NPs), and/or pharmacists, that have been increasingly adopted over time and may help address MO workload and geographic disparities.

## Figures and Tables

**Table 1:** Data sources.

Data Source	Acronym	Brief Description	Website
Canadian Institute for Health Information [11]	CIHI-SMDB	Contains information purchased from Scott’s Medical Database yearly. Demographic data collected from jurisdictional registrars, medical schools, the royal college of Canada and the college of family physicians of Canada. Each physician is uniquely identified over time. Data are for 1 January–31 December of the year of report	https://www.cihi.ca/en/scotts-medical-database-metadata (accessed on 16 November 2020)
Canadian Medical Association [9]	CMA-PDC	Draws information from members of the Canadian Medical Association using registration data. However, also contains data from the Royal College of Physicians and surgeons of Canada as well as the Collège des médecins du Québec. Actively audited data source. Data supplied are of 1 January of corresponding year.	https://www.cma.ca/physician-data-centre (accessed on 16 November 2020)
Canadian Post-M.D Education Registry [12]	CAPER	Supplied by the post-graduate medical education office of all training programs within the country on an annual basis. Includes residents and fellows training in Canada.Each year is the academic year Starting September of the listed year and running to the next year.	https://caper.ca/postgraduate-medical-education/annual-census (accessed on 16 November 2020)
Canadian Cancer Registry [13]	CCR	Government database on cancer incidence from all provincial and territorial cancer registries in Canada. Established in 1992 but contains data from the National Cancer Incidence Reporting system dating back to 1969.	https://www23.statcan.gc.ca/imdb/p2SV.pl?Function=getSurvey&Id=1535368 (accessed on 16 November 2020)
Statistics Canada		Federally run statistics organization.	https://www.statcan.gc.ca/ (accessed 22 January 2025)

**Table 2:** Comparison of annual incident malignancy cases to medical oncologist ratios between various different countries.

Country	Annual Incident Cancers per MO Provider
Austria ^a^	77
Hungary ^a^	79
Sweden ^a^	108
Italy ^a^	114
Finland ^a^	123
United States ^b^	137
Germany ^a^	146
Portugal ^a^	175
The Netherlands ^a^	229
Australia ^b^	272
Bulgaria ^a^	284
Belgium ^a^	304
Canada ^a^	354
France ^a^	416
United Kingdom ^a^	569

^a^ From De Azambuja E et al. [15], ^b^ Mathew, A [16].

## Data Availability

Data sources used in our analyses are outlined in Table 1.
